# Design and analysis of a terahertz metamaterial sensor with cross-shaped resonators for alkene detection

**DOI:** 10.1038/s41598-026-41228-5

**Published:** 2026-04-22

**Authors:** Esam Y. O. Zafar, Sayeeda Khanam, Ahmed Alqurashi, Mohammad Lutful Hakim, Zahriladha Zakaria, A. J. A. Al-Gburi

**Affiliations:** 1 Department of Electrical Engineering, College of Engineering and Architecture, Umm Al- Qura University, Makkah, 24382 Saudi Arabia; 2 Department of Electrical and Electronic Engineering, International Islamic University Chittagong (IIUC), Kumira, Chattogram, 4318 Sitakunda Bangladesh; 3 Department of Computer Science and Engineering, Southeast University, Dhaka, 1208 Bangladesh; 4 Center for Telecommunication Research & Innovation (CeTRI), Fakulti Teknologi Dan Kejuruteraan Elektronik Dan Komputer (FTKEK), Universiti Teknikal Malaysia Melaka (UTeM), Jalan Hang Tuah Jaya, Durian Tunggal, Melaka, 76100 Malaysia; 5 Strategic Research Institute (SRI), Asia Pacific University of Technology and Innovation (APU), Jalan Teknologi 5, Taman Teknologi Malaysia, Kuala Lumpur, 57000 Malaysia

**Keywords:** Terahertz (THz), Metamaterils, Cross-shaped resonator, Alkene, Detection, Absorber, Quality factor (Q), Engineering, Materials science, Optics and photonics, Physics

## Abstract

This paper presents the design and performance of a compact terahertz metamaterial absorber with strong angular stability and high sensing capability. The proposed structure, consisting of a fractal resonator fabricated on a polyamide substrate with copper metallization, achieves near-unity absorption (~ 99.99%) at 3.65 THz. The absorber demonstrates excellent angular robustness, maintaining stable resonance under TE polarization up to 60°, and preserving its primary mode under TM polarization despite the emergence of secondary resonances. At normal incidence, the structure also exhibits polarization insensitivity, ensuring consistent performance in practical excitation conditions. Unlike multiband meta-surfaces, which often suffer from complex resonance behavior and reduced resolution, the single-band design ensures a sharp, narrowband absorption peak, offering higher precision for detecting specific substances. Sensing performance is evaluated by monitoring resonance frequency shifts in response to changes in the surrounding refractive index. The absorber achieves a high sensitivity of 714.47 GHz/RIU, a figure of merit **(**FOM**)** of 14.29 RIU⁻¹, and a quality factor (**Q**) of 48.03, outperforming several previously reported terahertz absorbers while maintaining a compact footprint of 50 × 50 μm². The enhanced performance arises from strong electric field confinement and surface current localization within the fractal geometry, which amplifies light–matter interactions. Owing to its combination of high absorption efficiency, angular and polarization stability, and exceptional sensing performance, the proposed metamaterial absorber is a promising candidate for next-generation terahertz applications, including chemical and environmental sensing, and material characterization.

**Supplementary Information:**

The online version contains supplementary material available at 10.1038/s41598-026-41228-5.

## Introduction

 The monitoring and detection of hydrocarbons, particularly unsaturated hydrocarbons such as alkenes, is of great importance in petrochemical industries, fuel quality assessment, and environmental safety^1,2^. Alkenes such as octene, decene, hexene, and heptene are widely used in industrial applications, but their improper handling or undetected leakage can result in significant economic and safety concerns^3,4^. Thus, the study of reliable alkene detection technologies is essential to achieve accurate chemical monitoring and effective industrial process control. At present, conventional detection methods for hydrocarbons include gas chromatography–mass spectrometry (GC-MS)^5^, Fourier transform infrared spectroscopy (FTIR)^6^, and Raman spectroscopy^7^. Among these, GC-MS is most widely used, yet it requires complex sample preparation, chromatographic separation, and costly equipment. These procedures lead to cumbersome operations, long detection cycles, and difficulty in achieving real-time and in situ monitoring. Hence, it is essential to introduce a novel alkene monitoring technology that offers high precision, high signal-to-noise ratio, non-destructive measurement, and rapid response. Terahertz time-domain spectroscopy (THz-TDS) has emerged as a promising analytical tool due to its advantages of low photon energy, non-ionising radiation, high resolution, fast measurement, and rich spectroscopic information^8^. In recent years, researchers have explored the use of THz-TDS in chemical detection and hydrocarbon monitoring^9^. However, traditional THz-TDS methods face difficulties in tracing weak spectral features of alkenes and differentiating between closely related compounds, due to the inherently weak electromagnetic response of most hydrocarbons to terahertz waves^10^. To overcome this problem, high-performance metamaterial sensors operating in the terahertz band have attracted increasing research attention^11,12^. Metamaterials are artificially engineered composite structures with subwavelength unit resonators that exhibit electromagnetic properties beyond those of natural materials^13–15^. By exploiting resonant behaviors such as dipolar, LC, and lattice resonances, metamaterial absorbers can achieve strong field confinement and high sensitivity to the dielectric environment^16–18^. Absorption-based metamaterial designs are of particular interest because they can achieve near-perfect absorption at resonance frequencies, thereby enhancing detection stability and reducing measurement errors^19–22^. Recent advancements in metamaterial absorbers have significantly contributed to the sensing field. In^23^ a bi-tunable InAs-based absorber for thermal sensing with high absorption and magnetic tunability. A highly sensitive tunable terahertz absorber for biosensing applications^24^, and introduced a polarization-insensitive magnetically tunable absorber for refractive index sensing^25^. In^26^, developed a multi-resonant absorber for microorganism detection with high sensitivity and a wide frequency range for pesticide detection. a spoof surface plasmon-based metasensor for glucose and ethanol sensing, offering excellent sensitivity is presented in Haghverdi et al.^27^. In^14^, a wideband polarization conversion metasurface for cancer detection, a stable all-dielectric biosensor for hemoglobin measurement^12^, THz wave absorber for methane detection with enhanced refractive index sensitivity^28^. These studies demonstrate the versatility of metamaterial designs in chemical, biological, and environmental sensing.

Several studies have demonstrated the potential of metamaterial absorbers for chemical and liquid sensing. For example, Rabbani et al.^29^ proposed a triple-band absorber for oil identification, while Ge et al.^30^ reported a narrowband high-performance terahertz absorber with a Q-factor of 137 and sensitivity of 160 GHz/RIU. Wang et al.^31^ developed a biosensor based on elliptical ring resonators with high sensitivity, and Chen et al.^32^ achieved multimodal terahertz absorption for water–methanol mixtures with sensitivity up to 305 GHz/RIU. Forouzeshfard et al.^33^ proposed a semiconductor-based metamaterial sensor achieving 583 GHz/RIU sensitivity using plasmon-induced transparency. While these designs provide excellent sensitivity, most are limited to single or dual resonance peaks, which makes them vulnerable to external interference and restricts their robustness. Khan et al.^34^ designed a dual-band silicon ring resonator absorber with high Q-factors, while Zhang et al.^35^ introduced a polarization-insensitive dual-band absorber with refractive index sensitivities of 163 and 488 GHz/RIU, respectively. Nonetheless, research on alkene-specific detection using metamaterial absorbers remains scarce. Moreover, most reported designs focus on refractive index sensitivity, while fewer works investigate absorption performance and spectral depth as primary indicators of sensing ability.

To address these gaps, this work proposes a terahertz metamaterial absorption sensor with cross-shaped resonators specifically designed for alkene detection. The sensor exhibits a resonant frequency of 3.65 THz with 99.99% absorption efficiency. This strong and sharp absorption response enhances selectivity and sensitivity for detecting unsaturated hydrocarbons such as octene, decene, hexene, and heptene. Compared to conventional single-peak designs, the proposed sensor provides ultrahigh absorption, robustness against interference, and significant potential for real-time, nondestructive alkene monitoring in petrochemical and environmental applications.

## Design analysis

### Unit cell design

**Fig. 1:**
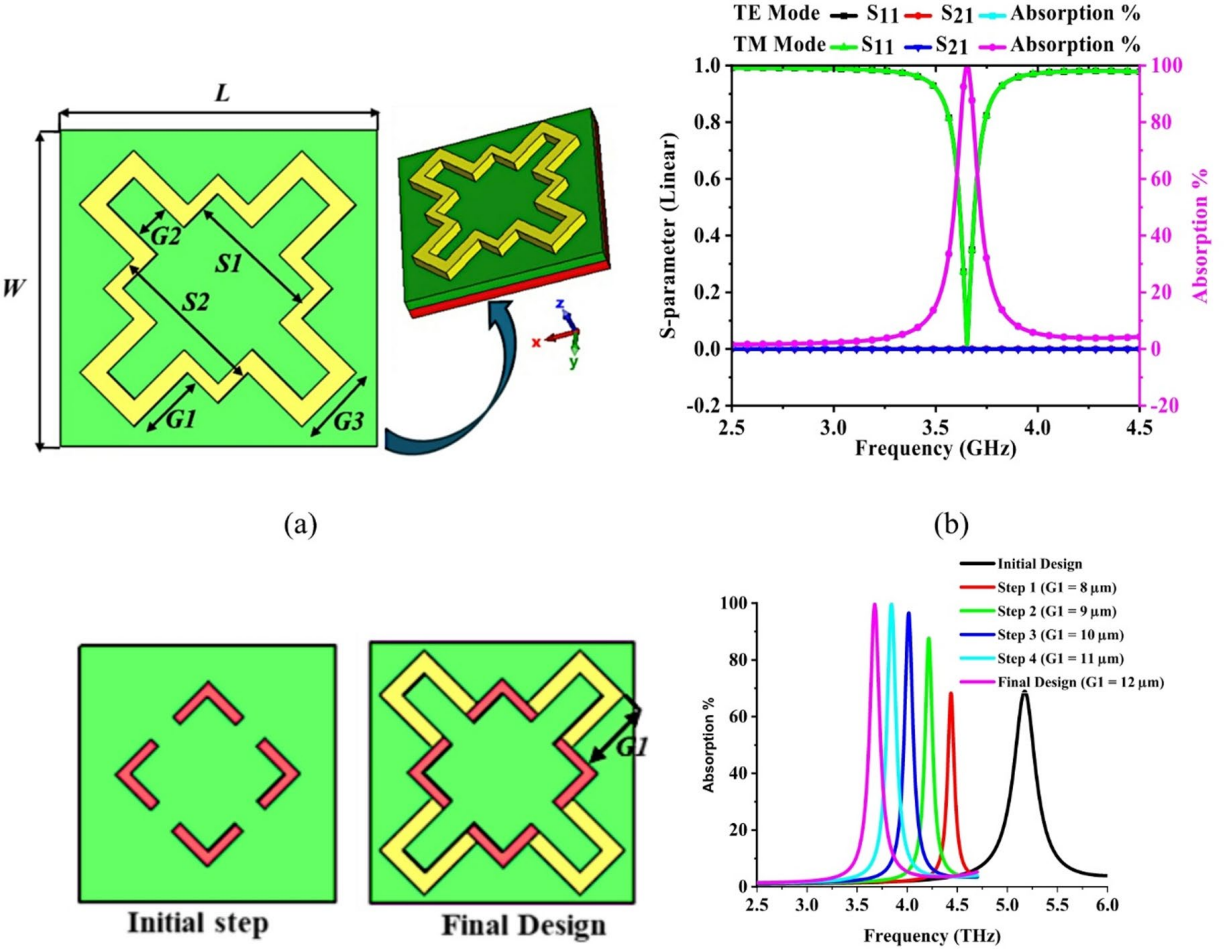
(**a**) Unit cell of the proposed terahertz absorber with a cross-shaped copper resonator on polyimide and aluminum ground. *G1* = 12 μm, *G2* = 4.0 μm, *G3* = 10 μm, *S1* = 16.97 μm, *S2* = 21.21 μm, *L* = 50 μm, and *W* = 50 μm. (**b**) Simulated spectra of the absorber showing reflection (S_11_), transmission (S_21_), and absorption. for both TE and TM mode, (**c**) Design evolution, and (**d**) absorption for different design steps.

The proposed terahertz metamaterial absorber consists of a cross-shaped copper resonator patterned on a polyimide dielectric spacer backed by a continuous aluminium ground plane, forming a typical metal–dielectric–metal (MDM) configuration^36^. Figure 1(a) shows the unit cell of the proposed terahertz absorber with a cross-shaped copper resonator on polyimide and aluminum ground. The unit cell, modelled in CST Microwave Studio, employs periodic boundary conditions along the x and y axes and a Floquet port excitation with a normally incident electromagnetic wave along the z axis. The top resonator is realized using annealed copper (σ = 5.8 × 107 S/m), the spacer is a lossy polyimide layer (ε_r_ = 3.5, tanδ = 0.0027), and the backplane is aluminium (σ = 3.56 × 107 S/m), ensuring zero transmission (S_21_≈0) and enabling perfect absorption through impedance matching. The thickness of copper, polyimide, and aluminium are 3.00 μm respactivily. The absorption spectrum A(*ω*) is calculated from the simulated reflection and transmission coefficients using Eq. (1)^36^.

1 $$A(\omega )=1 - \left| {S_{{11}}^{2}} \right| - \left| {S_{{21}}^{2}} \right|$$

Since the aluminum backplane suppresses transmission, the absorption simplifies to $A(\omega )=1 - \left| {S_{{11}}^{2}} \right|$. The resonant behavior arises from the *LC* interaction between the inductive currents along the copper arms and the capacitive gaps *G1​*,* G2​*,* G3*​, further enhanced by cavity coupling with the metallic ground. Parametric tuning reveals that increasing arm lengths increases inductance and redshifts the resonance, whereas enlarging gaps reduces capacitance and blueshifts it, while the polyimide thickness governs confinement and resonance linewidth. Figure 1(b) provides the simulated spectra of the absorber showing reflection (S_11_), transmission (S_21_), and absorption. for both TE and TM mode, a sharp absorption peak at 3.65 THz with a near zero reflection, negligible transmission, and a maximum absorption of 99.99%. The uniform results achived for both TE and TM mode due to the rotational symatric resonator design. The strong field confinement and perfect absorption at resonance confirm the device as a high-Q, narrowband absorber well-suited for alkene detection, where analyte-induced variations in refractive index or dielectric properties are expected to induce measurable frequency shifts and absorption changes, thus enabling selective and non-destructive hydrocarbon monitoring.

### Design Evolution Analysis

The design evolution of the unit cell is examined, highlighting the adjustments made to optimize performance. The design process progressed in multiple steps, each step refining the initial concept for better resonance characteristics and absorption efficiency. Figure 1(c) illustrates the design evolution, showing the progression from the initial structure to the final design. As shown in Fig. 1(d), the absorption spectra demonstrate how changes in the dimensions *G1* and resonator arm fractal length impact the absorption peak. Step 1 to Step 4 represent gradual adjustments made to the resonator dimensions, progressively improving the sharpness of the resonance. The effect of these adjustments can be explained using the resonant frequency formula (2)^13–15^

2 $$\:{f}_{0}=\frac{1}{2\pi\:\sqrt{LC}}$$

Where the inductance increases (*L*) as the arm lengths increase, causing a redshift in the resonance frequency. Similarly, as the gap sizes increase, the capacitance (*C*) decreases, which also shifts the resonance. This change in capacitance, due to varying gaps, contributes to the overall shift in the resonant frequency. In terms of quality factor, the sharpness of the resonance is quantified by Eq. (3)^13–15^, where $\:{f}_{0}$ is the resonant frequency and $\:{\Delta\:}f$is the resonance linewidth, which is the width of the resonance peak at half-maximum

3 $$\:Q=\frac{{f}_{0}}{{\Delta\:}f}$$

The final design, with an optimized cross-shaped resonator, exhibits a dramatic improvement in absorption efficiency, reaching nearly 100% at 3.65 THz, as seen in the comparison plot. This design evolution analysis highlights the importance of precise parameter tuning to achieve the desired performance in terms of sensitivity, resonance sharpness, and overall absorption.

### Equivalent circuit modelling

**Fig. 2:**
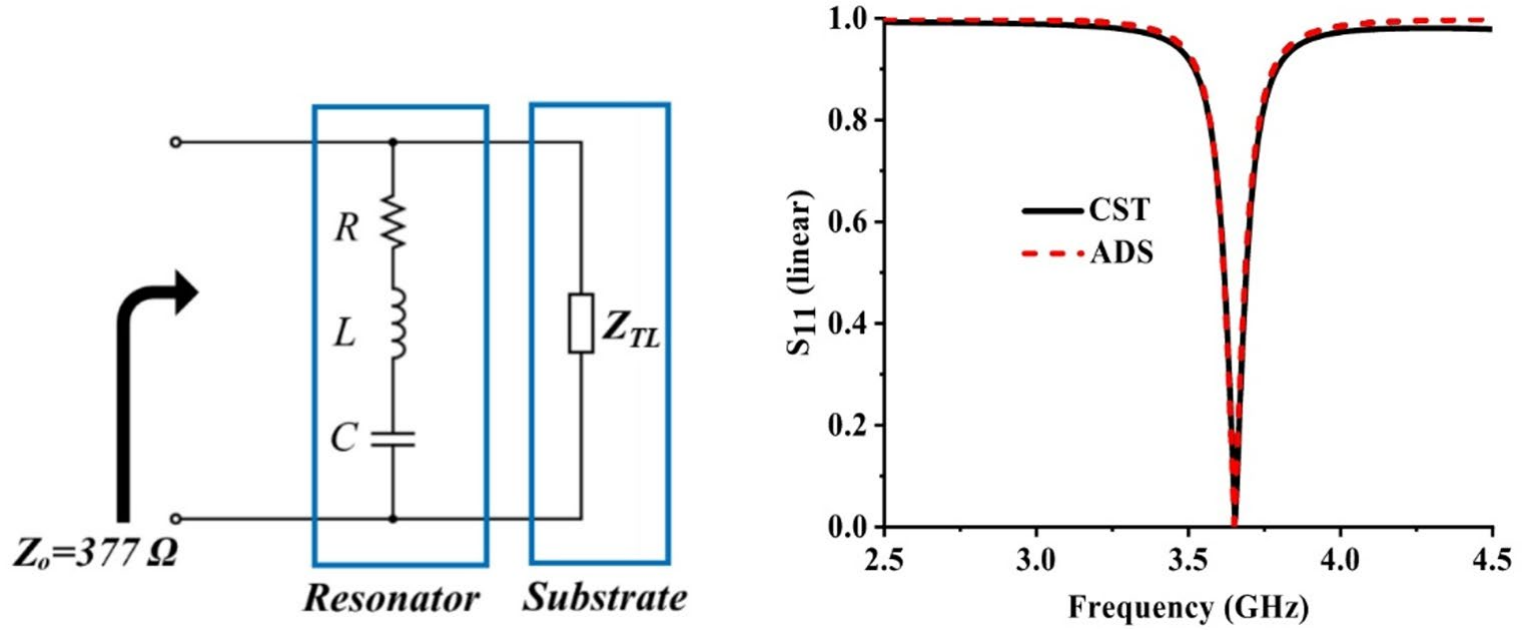
(**a**) Equivalent circuit model, and (**b**) S_11_ for circuit and EM simulation.

The equivalent circuit is modelled for proposed terahertz metamaterial absorber is shown in Figure (a) based on^18^ that consists of two main components: the *RLC* series circuit for the resonator, *Z*_*TL*_ **(**Transmission Line Model) for the substrate and *Z*_*0*_*= 377 Ω* is the imput impedence of free space. The resonator part of the absorber is represented by a series *RLC* circuit, where the resonant frequency $\:{f}_{0}$is given by the equation $\:{f}_{0}=\frac{1}{2\pi\:\sqrt{LC}}$. To achieve a resonant frequency of 3.65 THz, the inductance $\:L$ is chosen to be 1 nH for fractul resonator structure, and the corresponding capacitance $\:C$ is calculated to be 1.90 aF. The resistance $\:R$ in the circuit is adjusted to increment and decrement of the resonat depth. The Z_*TL*_ formed by the polyimide substrate, which is calculated to be 226.98 *Ω* based on the substrate’s dielectric constant $\:{\epsilon\:}_{r}=3.5$ by using Z_TL_=Zₒ/√ɛ. This impedance ensures optimal impedance matching between the resonator and the substrate. The *Z*_*TL*_ model represents the substrate’s behavior as a transmission line, which plays a crucial role in supporting the resonator’s performance and ensuring efficient energy transfer from the incident terahertz wave to the resonator. The designed equivalent circuit simulated using ADS (advance design softwer) and results well agree with the electromagnetic simulation shown in Fig. 2(b) confirming the design’s effectiveness. The calculated component values are *L* = 1 nH, *C* = 1.90 aF, and *R* = 300 Ω, ensuring maximum absorption and optimal performance for the proposed metamaterial absorber. The measured results can be obtain experimental setup and THz-TDS system as outlined in^1^.

### Mesh convergence

**Fig. 3:**
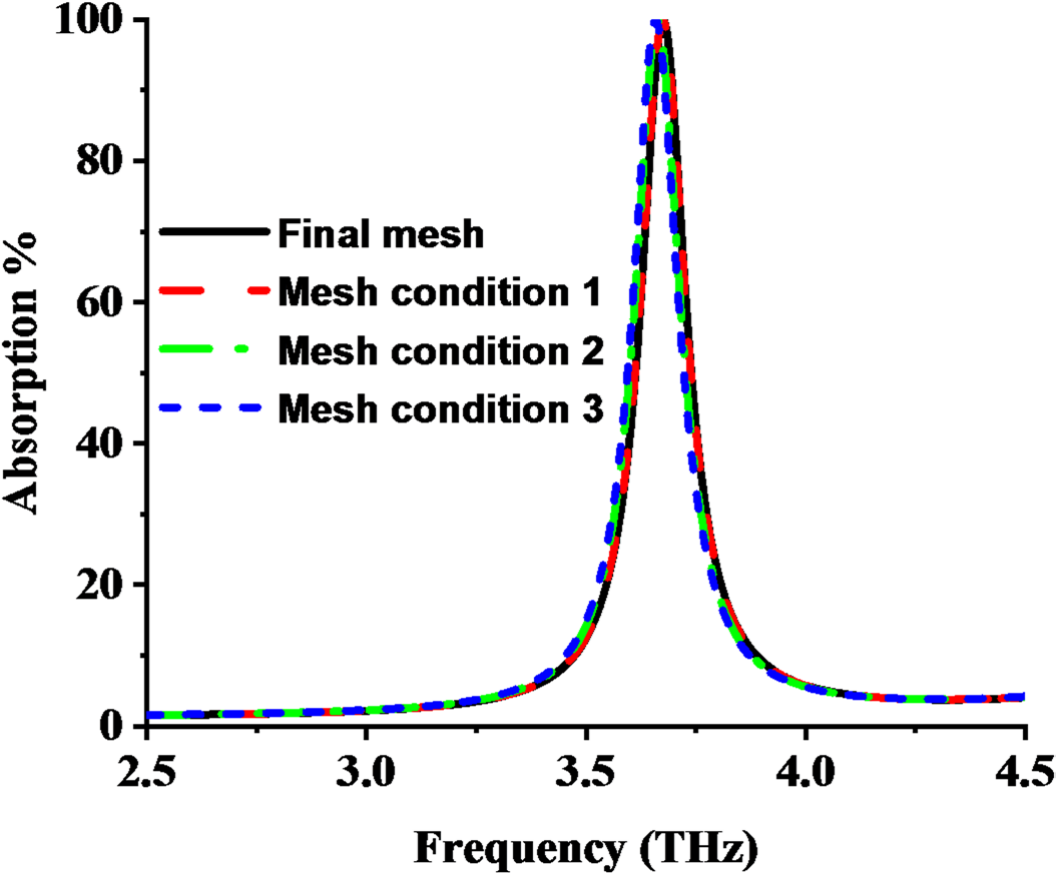
Mesh convergence study for absorption validation.

To ensure the reliability of the reported high absorption and Q-factor, a mesh convergence study is conducted using multiple mesh conditions. The simulation is initially performed with the default (automatic) mesh, which served as the final mesh with a minimum edge length of 0.118436, 34,726 tetrahedrons, and an average mesh quality of 0.745195. The mesh is progressively refined in Mesh Conditions 1, 2, and 3, where the cells per max model box edge were reduced from 10 (final mesh) to 8 (Condition 1), 6 (Condition 2), and 4 (Condition 3), with corresponding decreases in the minimum edge length and tetrahedrons count: 0.24 (Condition 1), 0.2208 (Condition 2), and 0.381473 (Condition 3). In Condition 1, the mesh contained 29,965 tetrahedrons, and the average mesh quality was 0.729851. In Condition 2, with 17,722 tetrahedrons, the average mesh quality was 0.728309, while in Condition 3, with 14,761 tetrahedrons, the average mesh quality was 0.714275. The absorption spectra from all mesh conditions were compared in Fig. 3, showing minimal variation in the absorption peak and resonance frequency up to Condition 3, indicating that the results had converged. This confirms that the simulation results are independent of further mesh refinement, validating the high absorption efficiency (~ 99.99%) and Q-factor (48.03). Therefore, the default mesh (final mesh) was considered sufficient for the simulations, providing a reliable and robust foundation for the performance of the proposed terahertz metamaterial absorber.

## Results and analysis

### Polarization insensitivity and incident angle stability

**Fig. 4:**
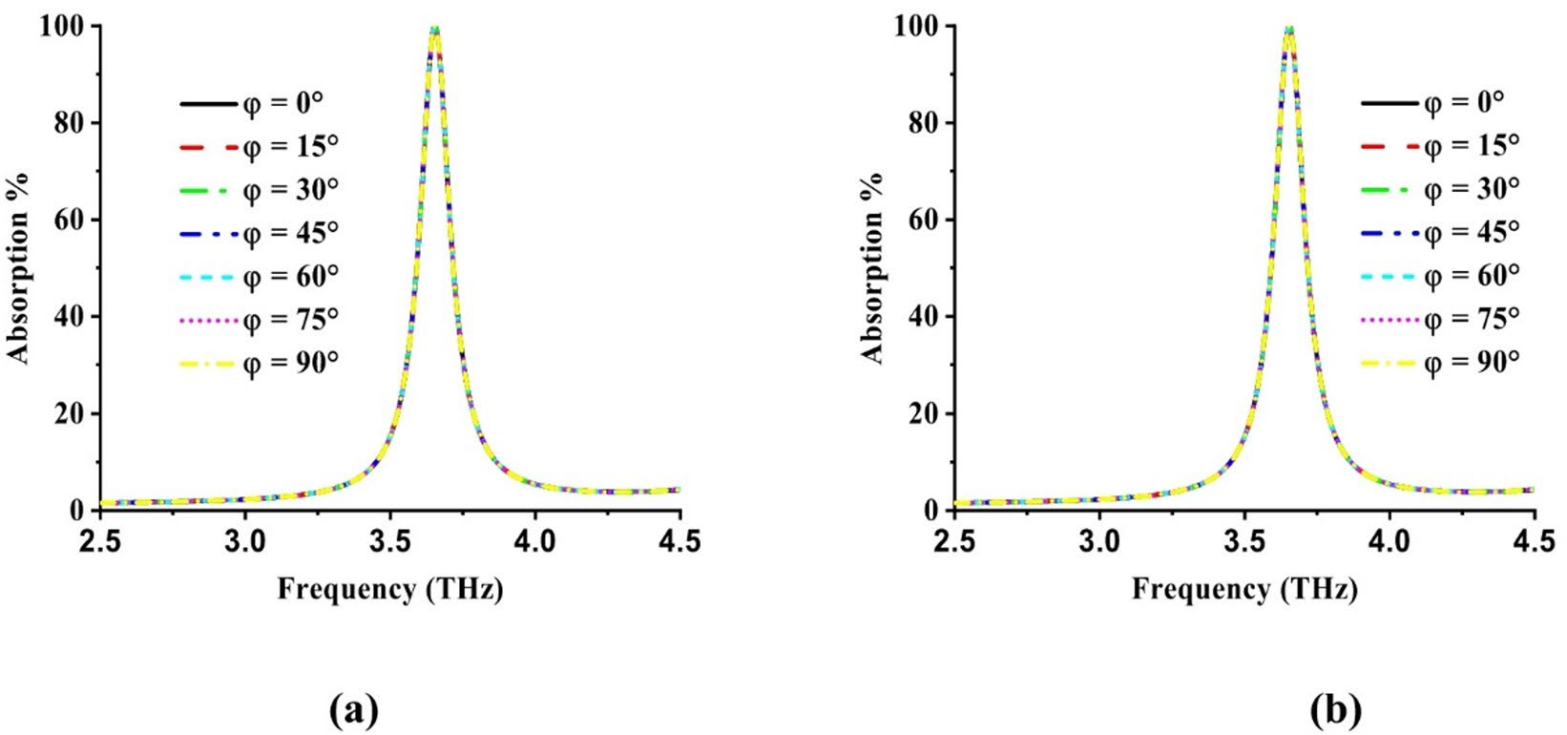
Absorption at different polarization angle for (**a**) TE and (**b**) TM mode.

The polarization dependence of the proposed cross-shaped resonator-based metamaterial absorber is assessed in both transverse electric (TE) and transverse magnetic (TM) modes^37^. As shown in Fig. 4(a) and Fig. 4(b), the absorption spectra for varying polarization angles exhibit minimal fluctuation in both modes. In the TE mode, where the electric field oscillates perpendicular to the plane of incidence, the absorption remains almost constant across a wide range of polarization angles. This is indicative of the sensor’s robustness to polarization changes, with the resonance frequency remaining stable and absorption efficiency reaching nearly 99.99%. Similarly, in the TM mode, where the electric field lies within the plane of incidence, the sensor demonstrates negligible shifts in its absorption characteristics regardless of the polarization angle. The sharp resonance peak at 3.65 THz with an absorption of 99.99% is maintained across all polarization orientations.

**Fig. 5:**
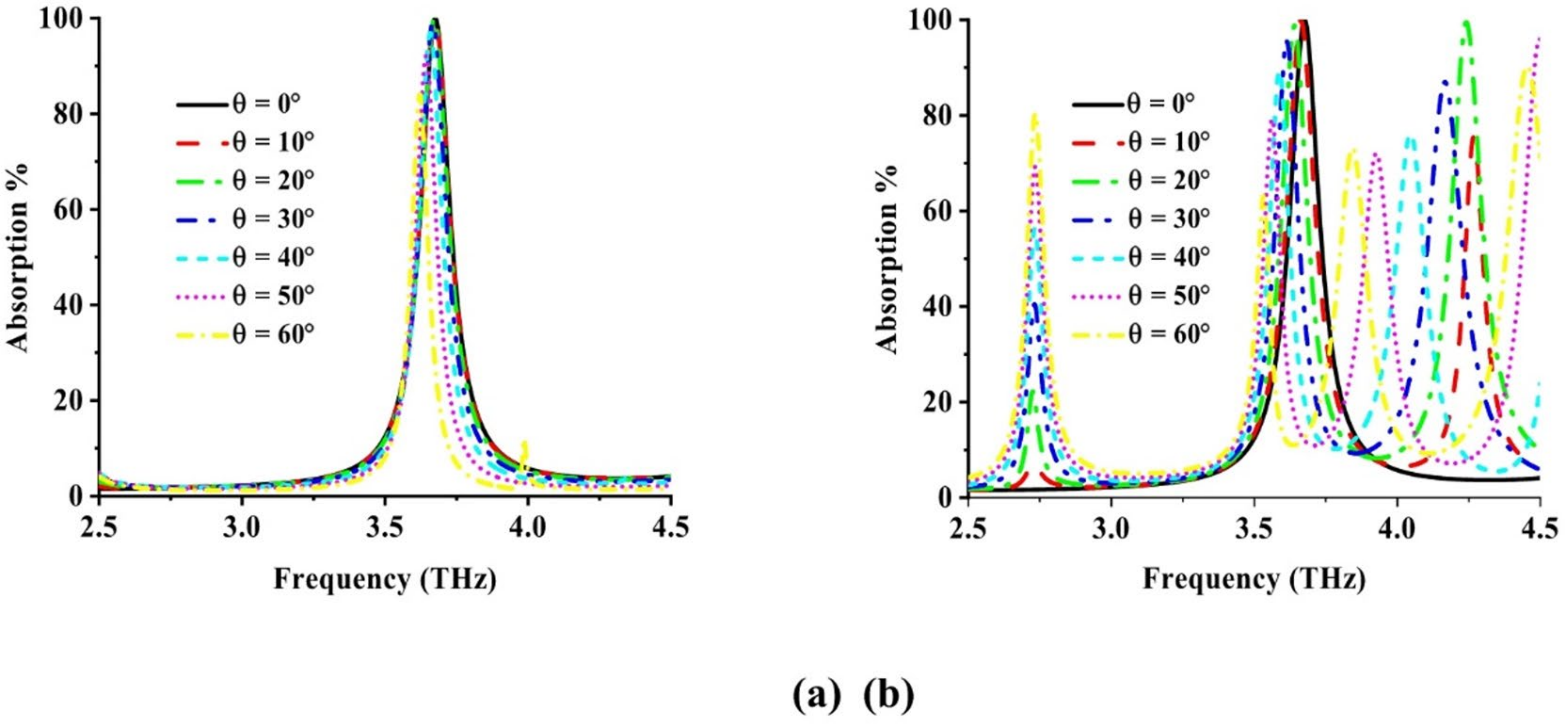
Absorption at different incident angle for (**a**) TE and (**b**) TM mode.

The absorption characteristics of the proposed terahertz metamaterial sensor were investigated for varying incident angles in both transverse electric (TE) and transverse magnetic (TM) modes^38^, as illustrated in Fig. 5. In the TE mode Fig. 5(a), the sensor demonstrates remarkable incident angle stability, with the primary absorption peak around 3.65 THz remaining sharp and almost invariant up to an incident angle of 60°. This indicates that the cross-shaped resonator design maintains strong electromagnetic confinement and consistent *LC* resonance behavior even under oblique incidence. The high absorption efficiency, approaching 99.99% at the resonance frequency, further confirms the robustness of the sensor against angular variation, making it suitable for practical scenarios where the incident THz wave may not always be perfectly normal. In contrast, the TM mode Fig. 5(b) exhibits more complex behavior, with additional secondary peaks emerging at higher frequencies as the incident angle increases. While the primary resonance at 3.65 THz remains relatively stable, the appearance of higher-order resonances in the TM mode can be attributed to the interaction between the magnetic field components and the resonator structure. In the TM mode, the electric field lies within the plane of incidence, leading to stronger coupling with the resonator’s metallic arms, which results in the formation of additional resonances at higher frequencies. These higher-order resonances become more pronounced under oblique incidence or due to the influence of the substrate on the overall resonant behavior. In contrast, the TE mode exhibits simpler resonance behavior, as the electric field is perpendicular to the plane of incidence. The interaction with the resonator in the TE mode is primarily electric, leading to fewer higher-order modes and a more straightforward resonance at the fundamental frequency. Despite these variations, the primary absorption peak remains pronounced across all tested angles, demonstrating the sensor’s resilience and potential for reliable detection even in non-ideal measurement conditions. Overall, these results confirm that the proposed sensor offers both polarization and angular insensitivity for the target resonance, which is a critical requirement for high-precision, real-time monitoring of alkenes in complex industrial and environmental environments.

### Surface current and field distribution

**Fig. 6:**
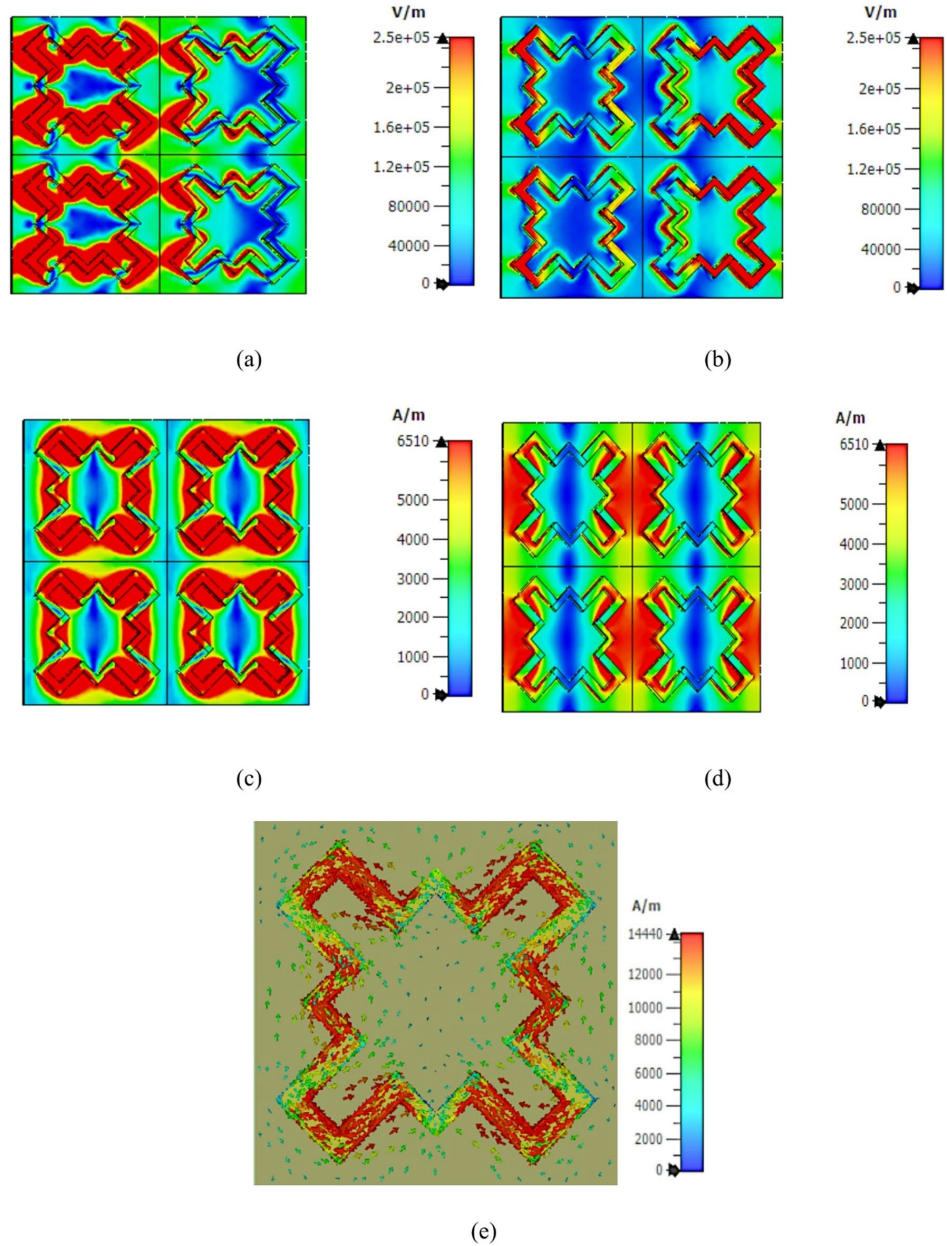
Electric field distribution of proposed absorber at 3.65 THz (**a**) TE, (**b**) TM, magnetic field distribution of proposed absorber at 3.65 THz (**c**) TE, (**d**) TM, and (**e**) Surface current distribution at 3.65 THz.

Figure 6 illustrates the electric and magnetic field distributions of the proposed terahertz metamaterial absorber at a resonance frequency of 3.65 THz^39,40^. In Figs. 6(a) and (b), the electric field distribution for the TE and TM modes is shown, respectively. For the TE mode Fig. 6(a), the electric field exhibits strong confinement around the resonator gaps, highlighting the effective electromagnetic interaction with the structure. In Fig. 6(b) for the TM mode, the electric field shows a different pattern, with polarization effects influencing the field distribution across the resonator arms. Moving to the magnetic field, Fig. 6(c) and Fig. 6(d) show the distribution for the TE and TM modes, respectively. In both cases, the magnetic field distribution further demonstrates the strong coupling between the resonator’s metallic structure and the incident terahertz waves. The field confinement and distribution patterns are crucial for understanding the resonator performance, particularly in terms of sensitivity and absorption efficiency. The sharp resonance and high absorption observed in the proposed sensor can be attributed to these strong field interactions. The surface current distribution at 3.65 THz is shown in the Fig. 6(e), which reveals the strong confinement of the electric field within the resonator structure. The current flows primarily along the metallic arms of the cross-shaped resonator, with the highest intensity concentrated at the resonator’s edges and corners. The current distribution is responsible for the resonant behavior of the absorber, where the electromagnetic field is effectively confined and localized. This results in a high absorption efficiency, as the surface current interacts strongly with the terahertz wave, leading to significant energy transfer between the material and the incident wave. The color map indicates the magnitude of the surface current in amperes per meter, demonstrating the regions of maximum and minimum current density across the resonator.

## Sensing Applications

**Fig. 7:**
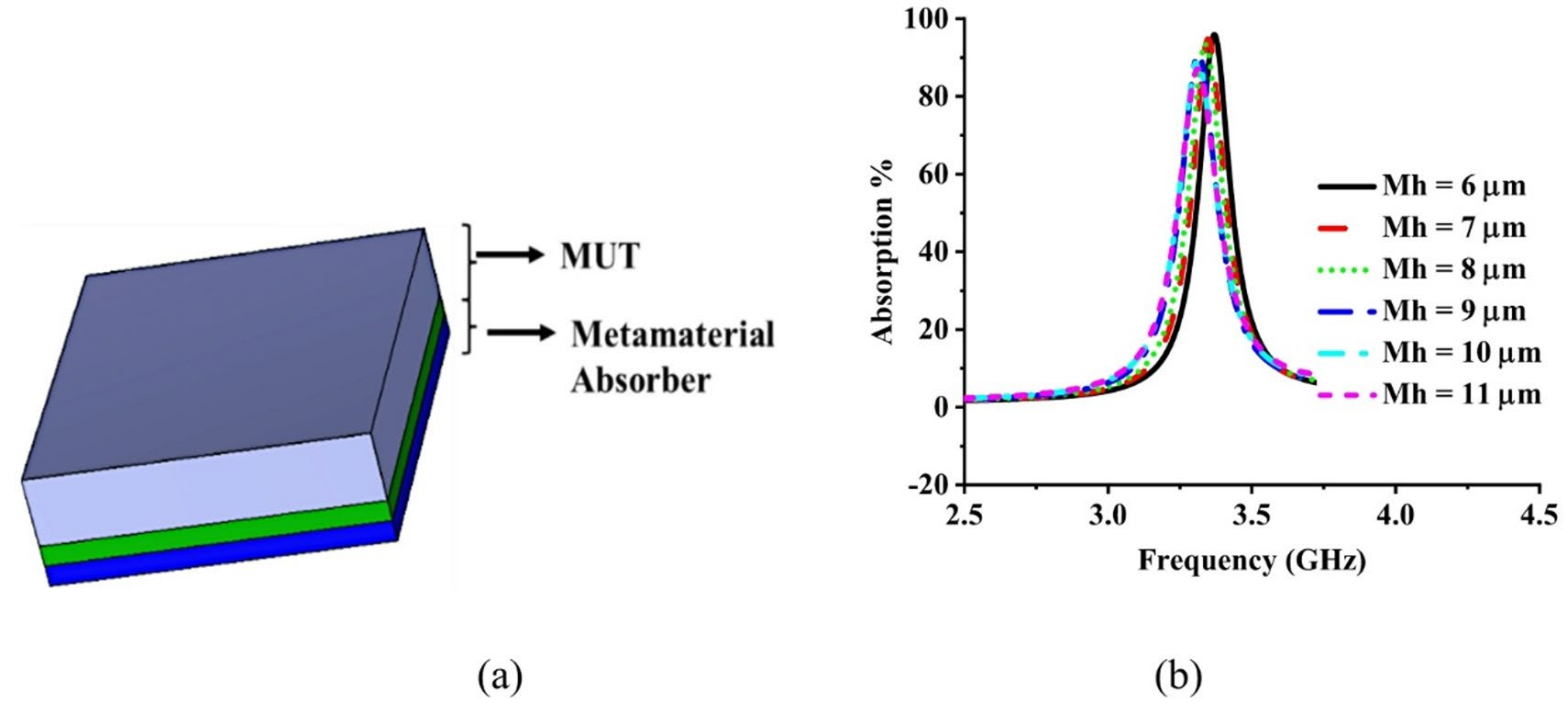
(**a**) sensor setup, and (**b**) MUT thickness investigation.

**Fig. 8:**
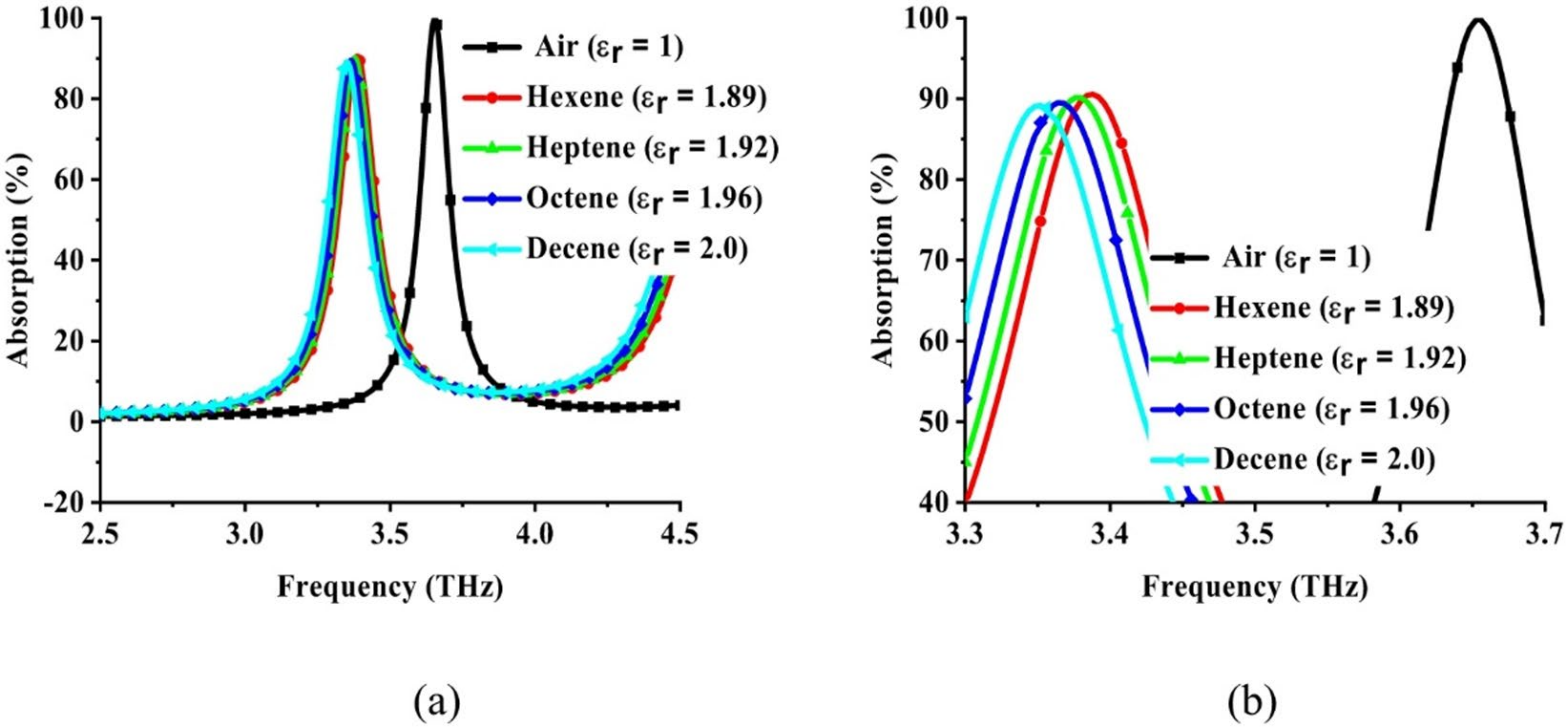
(**a**) Absorption plot for different permittivity value of MUT, (**b**) Zoomed view of peak resonance.

The designed terahertz metamaterial sensor, based on cross-shaped resonators, is investigated for its capability in alkene detection. In this sensing approach, the liquid analyte under test (MUT) is deposited on the resonator surface, as shown in Fig. 7 (a), thereby altering the effective dielectric environment. The relative permittivity of the MUT is assumed to be 2, and the thickness of the MUT, denoted as *Mh* is considered to be 10 μm. Initially, increasing the MUT thickness causes a shift in the resonance frequency. However, beyond 9 μm, the resonance frequency remains stable, indicating that the shift is no longer affected by the MUT thickness. As a result, a thickness of 10 μm is chosen for the MUT, where the resonant frequency shift is not influenced by further increases in the thickness. Figure 7(b) shows the MUT thickness investigation. Due to the strong confinement of the incident THz electromagnetic field around the resonator gaps, any change in the refractive index of the MUT leads to a perturbation of the resonance condition, resulting in a measurable frequency shift in the absorption spectrum shown in Fig. 8. This shift provides a reliable sensing mechanism for chemical identification. Table 1 lists the measured resonance frequencies corresponding to air, hexene, heptane, octene, and decene. As expected, the resonance frequency exhibits a progressive redshift as the refractive index increases from 1.000 to 1.414, confirming the strong dispersion-dependent interaction between the cross-shaped resonator and the MUT. The refractive index sensitivity S is defined in Eq. (4)^1^.

4 $$S=\frac{{\Delta f}}{{\Delta n}}$$

where *Δf* is the resonance frequency shift and *Δn* is the corresponding change in refractive index. Sensitivity quantifies the ability of the sensor to distinguish between analytes with similar refractive indices. In terms of practicality, sensitivity determines the precision with which the sensor can detect minute changes in the MUT’s dielectric properties. Expressing frequency in THz and refractive index in RIU, the sensitivity is converted into GHz/RIU by Eq. (5)^1^.

5 $$S[GHz/RIU]=\left( {\frac{{\Delta f}}{{\Delta n}}} \right) \times 1000$$

This parameter directly quantifies the ability of the sensor to distinguish between analytes with close refractive indices. While sensitivity provides a measure of frequency responsiveness, it does not account for resonance sharpness, which is essential for accurate analyte detection. The Figure of Merit (FOM) is defined by Eq. (6)^1^.

6 $$FOM=\frac{S}{{FWHM}}$$

where FWHM is the full width at half maximum of the resonance peak. A higher FOM indicates not only high sensitivity but also a narrow resonance linewidth, which implies better resolution and detection capability. The FOM is an important metric because it combines both the ability to detect miniature changes (sensitivity) and the clarity of detection (sharp resonance). To establish the quantitative relationship between resonance frequency f and refractive index *n*, a linear regression model is employed in Eq. (7)

7 $$f=a+b.n$$

where *a* is the intercept and *b* is the slope (sensitivity in THz/RIU). From the regression analysis, the coefficients are obtained as $\:a=4.36485\pm\:0.01066THz,\:\:\:\:b=-0.71447\pm\:0.00805THz/RIU$. Thus, the fit curve can be expressed by Eq. (8) shown in Fig. 8.

8 $$\hat {f}=4.36485 - 0.71447{{\cdot}}n$$

The negative slope reflects the redshift behavior, where the resonance frequency decreases with increasing refractive index. The residuals are calculated by $\:{e}_{i}={f}_{i}-{\widehat{f}}_{i}$. Where *f*_*i*_ is the measured frequency and $\:{\widehat{f}}_{i}$ is the fitted value. The Residual Sum of Squares (RSS) is calculated by Eq. (9).

9 $$RSS=\sum\limits_{{i=1}}^{N} {e_{i}^{2}} =2.426 \times 10_{{}}^{{ - 5}}THz_{{}}^{2}$$

The standard error of estimate is calculated by Eq. (10).

10 $${s_{e}}=\frac{{RSS}}{{N - 2}}=0.00284THz$$

These low residual values confirm the excellent fitting accuracy of the regression model, implying minimal error between the measured and predicted resonance frequencies. From the slope, the refractive index sensitivity is $\:S=-714.47\pm\:8.05GHz/RIU.$ Assuming a resonance linewidth of FWHM = 0.05 THz, the calculated figure of merit $\:FOM=\frac{\left|S\right|}{FWHM}=\frac{0.71447}{0.05}=14.29\pm\:0.16$. This FOM surpasses many previously reported THz sensors, which typically range between 5 and 12. The calculated FOM reflects the exceptional sharpness of the resonance, which is key to accurate chemical detection.

Finally, the results demonstrate that the proposed metamaterial sensor exhibits ultrahigh sensitivity (≈ 714 GHz/RIU) with a FOM of 14.29, placing it among the top-performing THz refractive index sensors. The near-perfect linearity ensures predictable and reproducible performance, which is crucial for practical chemical sensing. Compared to conventional planar resonator sensors, the cross-shaped design enhances electromagnetic confinement, leading to stronger interaction with analytes and sharper resonance peaks. In particular, the ability to distinguish between alkenes with very close refractive indices (hexene, heptene, octene, and decene) highlights the resolution power of the design. This capability is of significant interest for chemical analysis, biomedical diagnostics, and industrial monitoring in the terahertz regime. The excellent agreement between experimental data and regression analysis confirms that the sensor can serve as a reliable platform for quantitative dielectric characterization.

**Table 1:** Peak frequency for different permittivity/refractive index value of MUT.

MUT	Permittivity	Refractive index	Frequency (THz)
Air	1	1.000	3.65
Hexene	1.89	1.374	3.386
Heptene	1.92	1.385	3.377
Octene	1.96	1.400	3.364
Decene	2.0	1.414	3.351

**Fig. 9:**
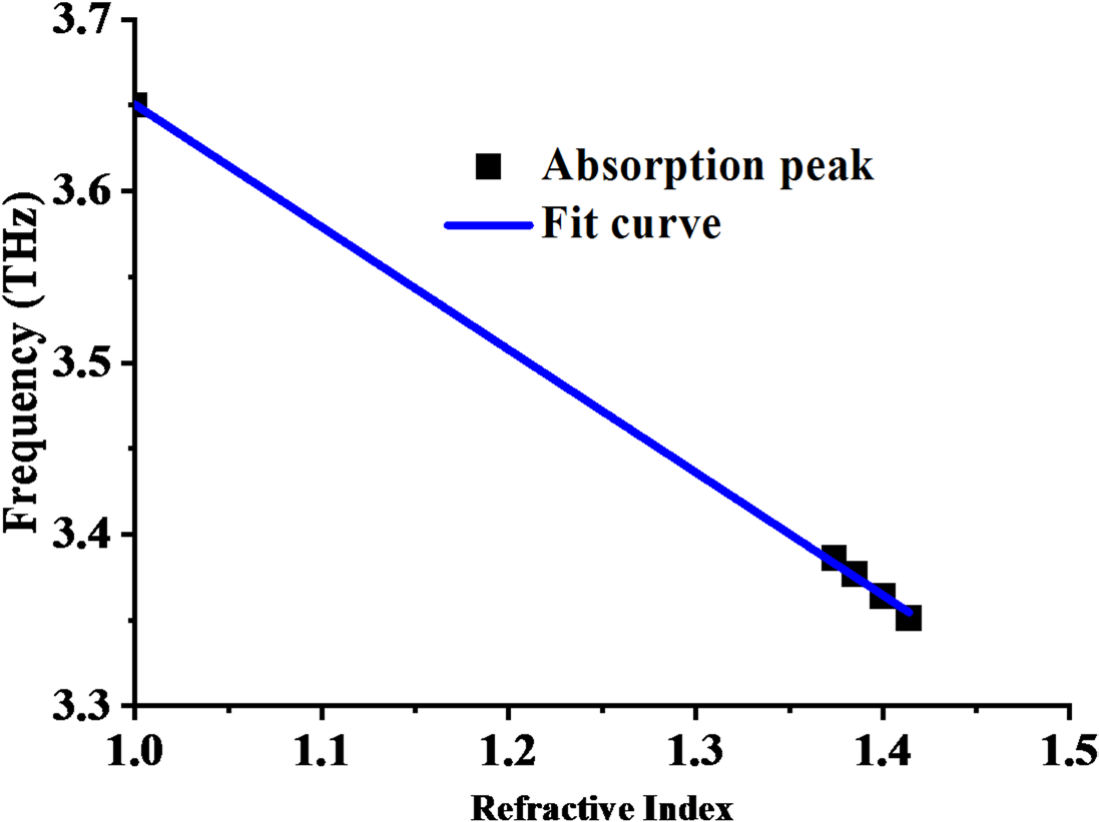
Fit curve for peak frequency.

## Comparison

The performance of the proposed terahertz metamaterial sensor has been systematically compared with several previously reported designs, as summarized in Table 2. The results clearly indicate that the proposed structure achieves superior sensing performance in terms of compactness, sensitivity, figure of merit (FOM), and quality factor (Q). The proposed 50 × 50 μm² fractal resonator design exhibits a significantly smaller footprint compared to the large-scale structures reported in^41^(84 × 84 μm²)^42^, (65 × 65 μm²)^43^, (100 × 100 μm²), and^44^(120 × 120 μm²). This high degree of miniaturization enhances field confinement and reduces material consumption while maintaining high resonance stability. The compact configuration also allows potential integration into on-chip and portable terahertz sensing systems, unlike the bulkier designs that require more complex and costly fabrication processes. In terms of material selection, the proposed sensor utilizes copper and polyamide, offering a low-cost and easily manufacturable alternative to the gold-based sensors in^41,44–46^. Although gold provides excellent conductivity, its high cost and limited fabrication scalability restrict its industrial adoption. Polyamide also offers mechanical flexibility and low dielectric loss, giving the proposed structure practical advantages over silicon-based^47^ and metal-only^48^ configurations, which may suffer from higher reflection losses and limited tunability. From an electromagnetic performance perspective, the operating frequency of the proposed sensor at 3.65 THz demonstrates an excellent balance between sensitivity and spectral resolution. This frequency closely aligns with^42^(3 and 6 THz) and^46^(1.09–4.06 THz), both of which operate in multi-band modes, yet the proposed single-band operation yields stronger resonance sharpness and reduced spectral overlap. Compared to^48^, which operates at a much higher range (5.972–7.934 THz), the proposed sensor provides a more practical frequency band for alkene detection, minimizing losses while retaining strong near-field coupling. The refractive index detection range of 1.0–1.41 makes the proposed sensor ideal for distinguishing among alkenes such as hexene, heptene, octene, and decene, while designs like^41,44,45^ operate over broader but less sensitive ranges (up to *n* = 2.6), leading to reduced precision for small analyte changes. Quantitatively, the Figure of Merit (FOM) of the proposed sensor is 14.29 RIU⁻¹, outperforming^41^(9.02 RIU⁻¹)^46^, (2.257 RIU⁻¹), and^47^(2.94 RIU⁻¹). This improvement corresponds to approximately 58% enhancement over^41^ and over sixfold improvement compared to^46^. The Quality Factor (Q) of 48.03 also exceeds that of^41^(Q = 32)^29^, (Q = 1.5), and^46^(Q = 13.89), highlighting the strong resonance selectivity and reduced energy loss of the proposed configuration. Meanwhile, the sensitivity reaches 714.47 GHz/RIU, which is significantly higher than those of^41^(285 GHz/RIU) and^47^(319.6 GHz/RIU), confirming its superior detection precision for even small refractive index variations. The reconfigurable U-shaped ring design in^43^ achieved a high Q-factor (Q = 86), but its limited sensing range (*n* = 1.0–1.03) and lower sensitivity (3.01 GHz/RIU) restrict its practical applicability for multi-analyte detection. Similarly, while^42^’s graphene-based disk resonator demonstrated excellent tunability and a high FOM of 11.75 RIU⁻¹, its dependence on graphene biasing and complex fabrication process increase cost and design complexity. In contrast, the proposed design achieves a higher FOM (14.29 RIU⁻¹) without requiring active control mechanisms, ensuring simpler realization. In summary, the proposed fractal terahertz metamaterial sensor offers a balanced and superior performance compared with existing works^41–48^. It combines miniaturized geometry, high FOM, enhanced Q-factor, and exceptional sensitivity with low-cost fabrication and polarization-insensitive operation. These unique advantages make it a promising candidate for real-time, label-free, and non-destructive sensing of organic compounds—particularly alkenes in chemical, biomedical, and environmental monitoring applications.

**Table 2:** Comparison with the existing works.

Ref. No	Size	Resonator Design	Materials Used	f (THz)	Range of *n*	FOM (RIU − 1)	Q	S (GHz/RIU − 1)
^41^	84 × 84	Metal–Graphene Hybrid	Gold, Graphene, Polyimide	0.1, 1.9	1.4–2.6	9.02	32	285
^42^	65 × 65	Graphene Disk Resonator	xxxx	3, 6	1.0–1.8	11.75	40.1	834
^45^	120 × 120	Symmetric SRR (Fano resonance)	Gold, Polyimide	1.267	1.0–2.0	–	1.5	40
^46^	40 × 40	Multiband Ring Resonator	Gold, Polyimide	1.09, 2.8, 4.06	1.0–2.45	2.257	13.89	514.28
^43^	100 × 100	Reconfigurable U-shaped Ring	Polyimide	3.045	1.0–1.03	--	86	3.01
^44^	120 × 120	Dual-band Square Ring Resonator	Gold, Polyimide	0.715, 1.013	1.0–2.0	--	--	152.1, 98.3
^47^	36 × 36	Complementary Split Ring Resonator (CSRR)	Gold, Silicon	2.249	1.35–1.39	2.94	22.1	319.6
^48^	200 × 200	All-Metal Hollow π-shaped	Metal	5.972–7.934	1.0–2.0	--	--	5.67–11.03
Proposed work	60 × 60	Fractal resonator	Copper, polyamide	3.65	1–1.41.41	14.29	48.03	714.47

## Conclusion

This paper proposed a compact terahertz metamaterial absorber based on a fractal resonator, designed on a polyamide substrate with copper as the conductive material. The structure achieves near-perfect absorption (~ 99.99%) at a resonance frequency of 3.65 THz and demonstrates strong angular and polarization stability, with TE mode performance maintained up to 60°. Polarization insensitivity at normal incidence further confirms the stability and practical reliability of the design. Beyond absorption, the proposed structure exhibits outstanding sensing performance. The refractive index sensing analysis shows a high sensitivity of 714.47 GHz/RIU, a figure of merit (FOM) of 14.29 RIU⁻¹, and a quality factor (Q) of 48.03. These values surpass many existing designs reported in the literature. The absorber achieves this performance within a compact unit cell size of 50 × 50 μm², which is significantly smaller than most comparable designs. The high sensitivity is attributed to strong electric field localization and surface current confinement within the fractal resonator arms, which enhances light–matter interaction. Overall, the proposed design combines high absorption efficiency, compactness, angular and polarization robustness, and exceptional sensing sensitivity, positioning it as a promising solution for next-generation terahertz technologies in biomedical diagnostics, chemical detection, and environmental monitoring, material characterization.

## Supplementary Information

Below is the link to the electronic supplementary material.

Supplementary Material 1

## Data Availability

The datasets used and/or analysed during the current study available from the corresponding author on reasonable request.

## References

[1] Y. Zhao, Z. Zhu, K. Zhao, Y. Zhao & C. Zhang, An insulating oil refractive index sensor based on multiband high-Q terahertz metamaterials. *IEEE Sens. J.* **24** (21), 34337–34346. 10.1109/JSEN.2024.3431696 (2024).

[2] Alqurashi, A., Khanam, S., Zafar, E. Y. & Al-Gburi, A. J. A symmetric dual-ring cross stub based dual-band THz metamaterial absorber design for permittivity sensing applications. *Results Opt.* 100917. 10.1016/j.rio.2025.100917 (2025).

[3] Zhang, Y., Zhou, Z. L., Li, J. H. & Li, Y. T. Electrochemical difunctionalization of alkenes. *Chem. Record*. **25** (6), e202400263 (2025). 39901507 10.1002/tcr.202400263

[4] Jacobs, T. L. The Chemistry of Alkenes. *J. Am. Chem. Soc.* **88** (10), 2350–2351 (1966).

[5] Meira, M., Verucchi, C., Álvarez, R. & Catalano, L. Dissolved gas analysis in mineral oil and natural ester liquids from thermal faults. *IEEE Trans. Dielectr. Electr. Insul.* **28** (4), 1317–1325 (2021).

[6] Bakar, N. A. & Abu-Siada, A. A new method to detect dissolved gases in transformer oil using NIR-IR spectroscopy. *IEEE Trans. Dielectr. Electr. Insul.* **24** (1), 409–419 (2017).

[7] Song, R., Chen, W., Wang, Y., Du, L. & Wang, P. Transformer aging diagnosis method based on Raman spectroscopy wavelet packet-SPCA feature extraction. *IEEE Trans. Instrum. Meas.* **72**, 1–8 (2022).

[8] Sun, X., Cui, D., Shen, Y., Li, W. & Wang, J. Non-destructive detection for foreign bodies of tea stalks in finished tea products using terahertz spectroscopy and imaging. *Infrared Phys. Technol.* **121**, 104018 (2022).

[9] He, Y. et al. A new method for detecting trace methanol in insulating oil based on terahertz spectroscopy, in *International Conference on Electrical Materials and Power Equipment (ICEMPE)*, 2021: IEEE, 1–4., 2021: IEEE, 1–4. (2021).

[10] Gu, H., Shi, C., Wu, X. & Peng, Y. Molecular methylation detection based on terahertz metamaterial technology, *Analyst*, **145** (20), 6705–6712, (2020). 10.1039/d0an01062f 32812556

[11] Ahmed, K. et al. Refractive index-based blood components sensing in terahertz spectrum. *IEEE Sens. J.* **19** (9), 3368–3375 (2019).

[12] Nagini, K. S. & Chandu, D. Highly sensitive and angular stable all-dielectric cross-polarization conversion based biosensor, in *2023 IEEE MTT-S International Microwave Biomedical Conference (IMBioC)*, : IEEE, 190–192. (2023).

[13] Hakim, M. L. et al. Quad-band polarization-insensitive square split-ring resonator (SSRR) with an inner Jerusalem cross metamaterial absorber for Ku-and K-Band sensing applications, *Sensors*, **22** (12), 4489, (2022). 10.3390/s22124489 PMC9228148 35746277

[14] Gupta, A., Kumar, V., Garg, S. B., Bansal, S. & Al-Gburi, A. J. Graphene-based terahertz antenna with enhanced backscatter sensitivity for early breast cancer localization. *Nano Trends* **12**, 100169. 10.1016/j.nwnano.2025.100169 (2025).

[15] Zhao, C., Wang, H., Bu, Y., Zou, H. & Wang, X. Structures, principles, and properties of metamaterial perfect absorbers. *Phys. Chem. Chem. Phys.* **25** (44), 30145–30171 (2023). 37916298 10.1039/d3cp03346e

[16] Kumar, S. et al. Highly sensitive, selective and portable sensor probe using germanium-doped photosensitive optical fiber for ascorbic acid detection. *IEEE Sens. J.* **21** (1), 62–70 (2020).

[17] Shadab, A., Raghuwanshi, S. K. & Kumar, S. Advances in micro-fabricated fiber Bragg grating for detection of physical, chemical, and biological parameters—A review. *IEEE Sens. J.* **22** (16), 15650–15660 (2022).

[18] Nagini, K. S. & Chandu, D. Terahertz wideband cross-polarization conversion metasurface based on double split-ring resonator. *AEU-International J. Electron. Commun.* **170**, 154826 (2023).

[19] Islam, M. R. et al. Star enclosed circle split ring resonator-based metamaterial sensor for fuel and oil adulteration detection. *Alexandria Eng. J.* **67**, 547–563 (2023).

[20] Abdolrazzaghi, M., Kazemi, N., Nayyeri, V. & Martin, F. AI-assisted ultra-high-sensitivity/resolution active-coupled CSRR-based sensor with embedded selectivity, *Sensors*, **23** (13), 6236, (2023). 10.3390/s23136236 PMC10347157 37448086

[21] Hakim, M. L. et al. Polarization insensitive symmetrical structured double negative (DNG) metamaterial absorber for Ku-band sensing applications. *Sci. Rep.* **12** (1), 479 (2022). 35013437 10.1038/s41598-021-04236-1 PMC8748699

[22] Li, Z. et al. Actively tunable multi-frequency narrowband terahertz absorber using graphene metamaterials. *Opt. Commun.* **583**, 131768 (2025).

[23] Niharika, N. & Singh, S. Theoretical analysis of InAs based Bi-tunable narrow band terahertz perfect absorber for thermal sensing application. *Micro Nanostruct.* **194**, 207936 (2024).

[24] Niharika, N. & Singh, S. Highly sensitive tunable terahertz absorber for biosensing applications, *Optik*, **273**, 170476, (2023).

[25] Niharika, N. & Singh, S. Polarization-insensitive magnetically tunable perfect absorber for refractive index sensing applications. *Int. J. Mod. Phys. B*. **38** (29), 2450399 (2024).

[26] Bhati, R. & Malik, A. K. Multiband terahertz metamaterial perfect absorber for microorganisms detection. *Sci. Rep.* **13** (1), 19685 (2023). 37952035 10.1038/s41598-023-46787-5 PMC10640598

[27] Bhati, R., Jewariya, M. & Malik, A. K. Spoof surface plasmon-based terahertz metasensor for glucose and ethanol. *Appl. Phys. A*. **128** (9), 840 (2022).

[28] Haghverdi, A. B., Rezaei, I., Khani, A. A. M. & Aghaee, T. Methane detection approach based on THz wave absorber. *Sens. BioSensing Res.* **47**, 100758. 10.1016/j.sbsr.2025.100758. (2025).

[29] Rabbani, M. G. et al. Orthogonal centre ring field optimization triple-band metamaterial absorber with sensing application. *Eng. Sci. Technol. Int. J.* **49**, 101588 (2024).

[30] Ge, H. et al. Design of high-performance terahertz sensor based on metamaterials, in *Journal of Physics: Conference Series*, 2174 (1): IOP Publishing, 012001. (2022).

[31] Wang, Z., Geng, Z. & Fang, W. Exploring performance of THz metamaterial biosensor based on flexible thin-film. *Opt. Express*. **28** (18), 26370–26384 (2020). 32906910 10.1364/OE.402222

[32] Chen, M. et al. Terahertz sensing of highly absorptive water-methanol mixtures with multiple resonances in metamaterials. *Opt. Express*. **25** (13), 14089–14097 (2017). 28788994 10.1364/OE.25.014089

[33] Forouzeshfard, M. R., Ghafari, S. & Vafapour, Z. Solute concentration sensing in two aqueous solution using an optical metamaterial sensor. *J. Lumin.* **230**, 117734 (2021).

[34] Khan, M. S., Varshney, G. & Giri, P. Altering the multimodal resonance in ultrathin silicon ring for tunable THz biosensing. *IEEE Trans. Nanobiosci.* **20** (4), 488–496 (2021). 10.1109/TNB.2021.3105561 34410927

[35] Zhang, H. et al. A dual-band terahertz metamaterial sensor with high Q-factor and sensitivity. *Opt. Quant. Electron.* **55** (13), 1126 (2023).

[36] Hakim, M. L. et al. Ultrawideband polarization-independent nanoarchitectonics: a perfect metamaterial absorber for visible and infrared optical window applications, *Nanomaterials*, vol. **12** (16), 2849, (2022). 10.3390/nano12162849 PMC9412529 36014711

[37] Hakim, M. L., Islam, M. T. & Alam, T. Ultra-miniaturized conformal polarization insensitive and incident angle stable FSS for n257 band 5G EMI shielding applications. *IEEE Trans. Antennas Propag.* **72** (10), 7905–7915 (2024).

[38] Hakim, M. L. et al. Triple-band square split-ring resonator metamaterial absorber design with high effective medium ratio for 5G sub-6 GHz applications, *Nanomaterials*, **13** (2), 222, (2023). 10.3390/nano13020222 PMC9861934 36677975

[39] Shao, L. et al. Graphene terahertz metamaterials absorber with multiple absorption peaks and adjustable incident polarization angle. *Physica B: Condens. Matter* **714**, 417427. 10.1016/j.physb.2025.417427 (2025).

[40] Shruti, S., Pahadsingh & Appasani, B. Metamaterial-based terahertz absorbers for refractive index sensing: types, mechanism, and applications, *Plasmonics*, **20**(7), 5557–5571, (2025).

[41] Zhang, D. Dynamically tunable terahertz metamaterial sensor based on metal–graphene hybrid structural unit. *AIP Adv.* **12** (2), 025206. 10.1063/5.0079964 (2022).

[42] Rezagholizadeh, E., Biabanifard, M. & Borzooei, S. Analytical design of tunable THz refractive index sensor for TE and TM modes using graphene disks. *J. Phys. D*. **53** (29), 295107 (2020).

[43] Shruti, S. et al. A reconfigurable terahertz metamaterial absorber for gas sensing applications, *Crystals*, vol. 13, no. 2, p. 158, (2023).

[44] Wang, D. et al. A high Q-factor dual-band terahertz metamaterial absorber and its sensing characteristics, *Nanoscale*, **15**(7), 3398–3407, (2023). 10.1039/d2nr05820k 36722909

[45] Al-Naib, I. Thin-film sensing via fano resonance excitation in symmetric terahertz metamaterials. *J. Infrared Millim. Terahertz Waves*. **39** (1), 1–5 (2018).

[46] Veeraselvam, A., Mohammed, G. N. A., Savarimuthu, K. & Vijayaraman, P. D. An ultra-thin multiband refractive index-based carcinoma sensor using THz radiation. *IEEE Sens. J.* **22** (3), 2045–2052 (2021).

[47] Saadeldin, A. S., Hameed, M. F. O., Elkaramany, E. M. & Obayya, S. S. Highly sensitive terahertz metamaterial sensor. *IEEE Sens. J.* **19** (18), 7993–7999 (2019).

[48] Banerjee, S. et al. All-Metal Metamaterial-Based Sensor with Novel Geometry and Enhanced Sensing Capability at Terahertz Frequency, *Sensors*, **25**(2), 507, (2025). 10.3390/s25020507 PMC11769316 39860877

