# Supplementary material for: Design and analysis of a terahertz metamaterial sensor with cross-shaped resonators for alkene detection
Source: Sci Rep. 2026 Apr 22;16:18781. doi: 10.1038/s41598-026-41228-5 (PMC13272807; doi:10.1038/s41598-026-41228-5)
Supplement: Supplementary file 1 — Supplementary Material 1 [file 41598_2026_41228_MOESM1_ESM.pdf]

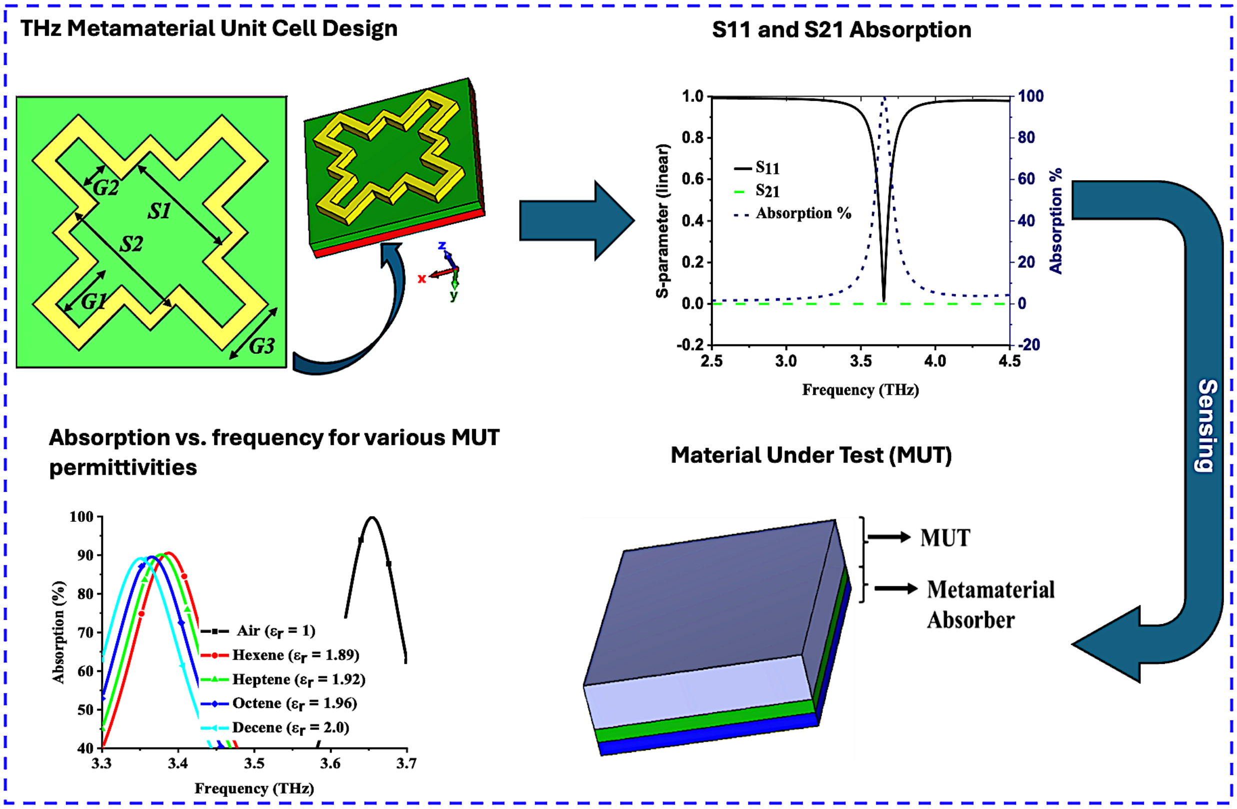
THz Metamaterial Unit Cell Design
G2
S1
S2
G1
G3
z
x
y
S11 and S21 Absorption
S-parameter (linear)
Absorption %
S11
S21
Absorption %
1.0
0.8
0.6
0.4
0.2
0.0
-0.2
100
80
60
40
20
0
-20
2.5
3.0
3.5
4.0
4.5
Frequency (THz)
Sensing
Absorption vs. frequency for various MUT permittivities
Absorption (%)
100
90
80
70
60
50
40
3.3
3.4
3.5
3.6
3.7
Frequency (THz)
Air (εr = 1)
Hexene (εr = 1.89)
Heptene (εr = 1.92)
Octene (εr = 1.96)
Decene (εr = 2.0)
Material Under Test (MUT)
MUT
Metamaterial Absorber
